# Clear cell sarcoma of the scapula. A case report and review of the literature

**DOI:** 10.1186/1477-7819-4-48

**Published:** 2006-08-07

**Authors:** Constantinos J Kazakos, Vasilios G Galanis, Alexandra Giatromanolaki, Dennis-Alexander J Verettas, Efthimios Sivridis

**Affiliations:** 1 Department of Orthopedics, University Hospital of Alexandroupolis, Democritus University of Thrace, Alexandroupolis, Greece; 2 Department of Pathology, University Hospital of Alexandroupolis, Democritus University of Thrace, Alexandroupolis, Greece

## Abstract

**Background:**

Clear cell sarcoma of tendons and aponeuroses (CCSTA) appears usually in the extremities and rarely in the trunk.

**Case presentation:**

We present an unusual case of CCSTA overlying the scapular region and with secondary osseous extension in the lower scapula. The patient underwent a wide local excision with removal of the tumor and the lower two thirds of the scapula. He had no local recurrences but he developed lung metastases after 5 months in spite of postoperative chemotherapy. He finally died ten months later.

**Conclusion:**

The patients with CCSTA have a variable unpredictable course. Despite treatment the overall prognosis is poor.

## Background

Clear cell sarcoma of tendons and aponeuroses (CCSTA), first described by Enzinger in 1965[1], is a rare tumor accounting for less than 1% of all soft tissue sarcomas [2]. It has a clear distinction from metastatic melanoma. Patients with CCSTA have a variable unpredictable prognosis. This tumor has a predilection for the lower and upper limbs and rarely presents in the trunk. Sporadic cases only have been reported of CCSTA involving primarily the bone or extending from soft tissues to surrounding bones. We report an unusual case of CCSTA in the left lower scapular region with secondary osseous invasion of the scapula.

## Case presentation

A 61-year-old man presented with a painful mass overlying the lower left scapula of four months duration. His pain deteriorated the last three weeks and was accompanied by limitation of the range of motion of the left glenohumeral joint.

Physical examination revealed a subcutaneous tender uniformly firm mass in the lower left scapular region measuring 4.5 × 4.5 cm in diameter. There were no signs of superficial skin inflammation. The patient had not a previous history of melanoma and/or other skin tumors.

**Figure 1:**
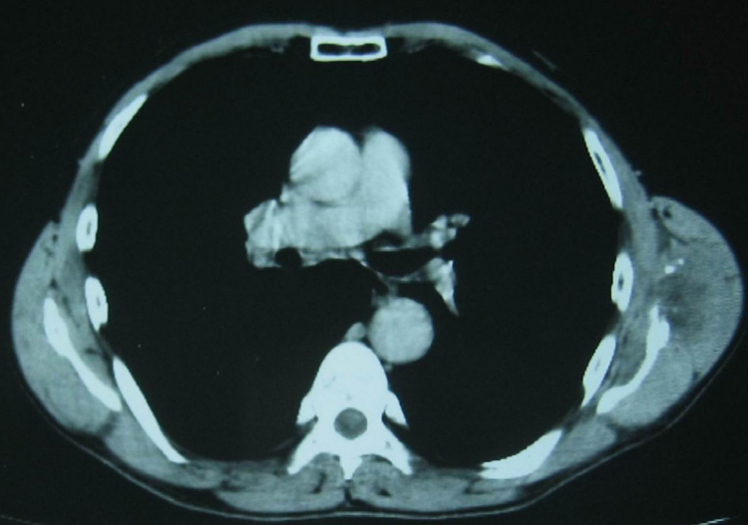
Axial CT scan revealed a soft tissue mass localized in the lower scapular region with intralesional calcifications and a secondary osseous invasion of the lower scapula.

**Figure 2:**
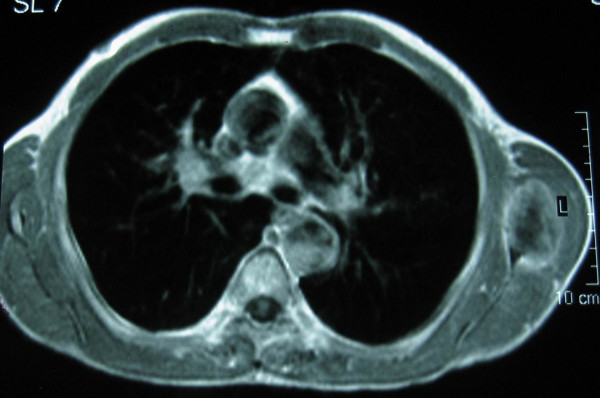
MRI image established the tumor measuring 45 × 45 mm.

**Figure 3:**
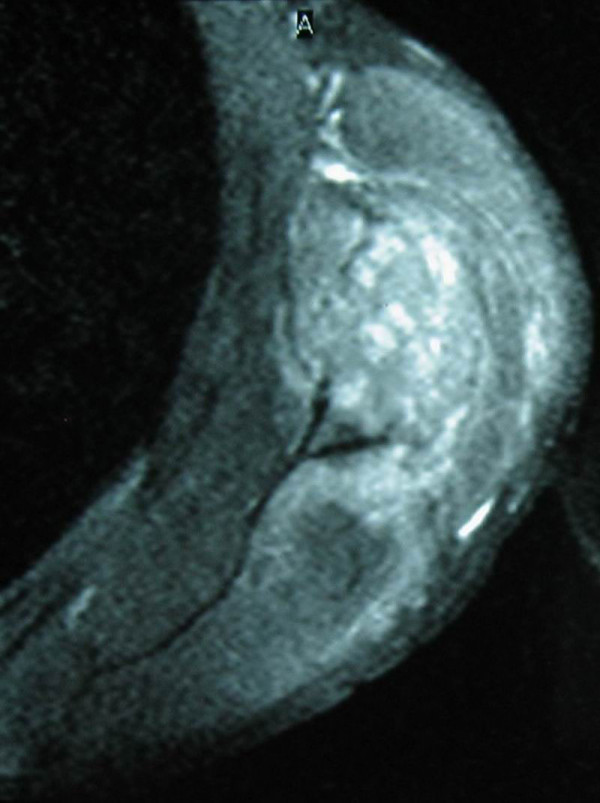
Saggital MRI image through the scapular region showed the soft tissue tumor.

Radiological examination showed an osteolytic lesion in the lower left scapula while bone scanning was positive with increased uptake in the same area. CT scans and MRI images of the thorax and shoulder showed a soft tissue mass 45 × 45 mm localized in the lower scapular region between infraspinatus and teres minor muscles with bone invasion of the lower part of the scapula (Figure 1, 2, 3). There were no lung metastases neither axillary nor mediastinal lymph nodes.

**Figure 4:**
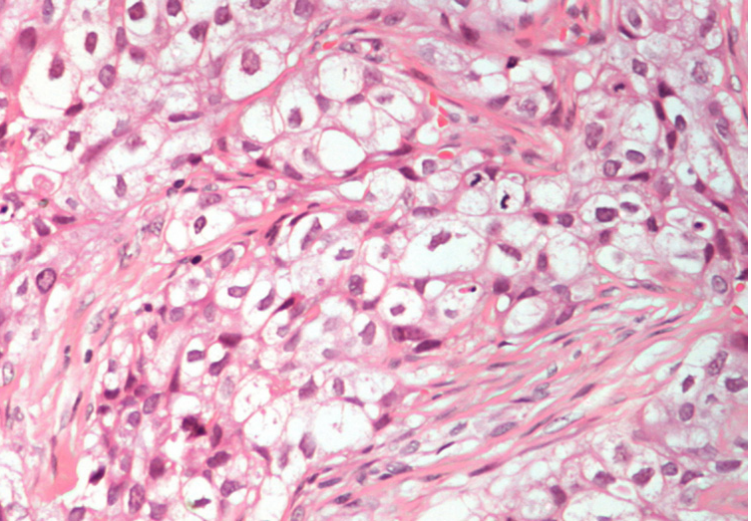
Clear cell sarcoma tissue section: compact nests and fascicles with large rounded and spindle-shaped cells separated by connective tissue septa. Mitoses are numerous. The cells had large amounts of clear cytoplasm (*H&E *×200).

**Figure 5:**
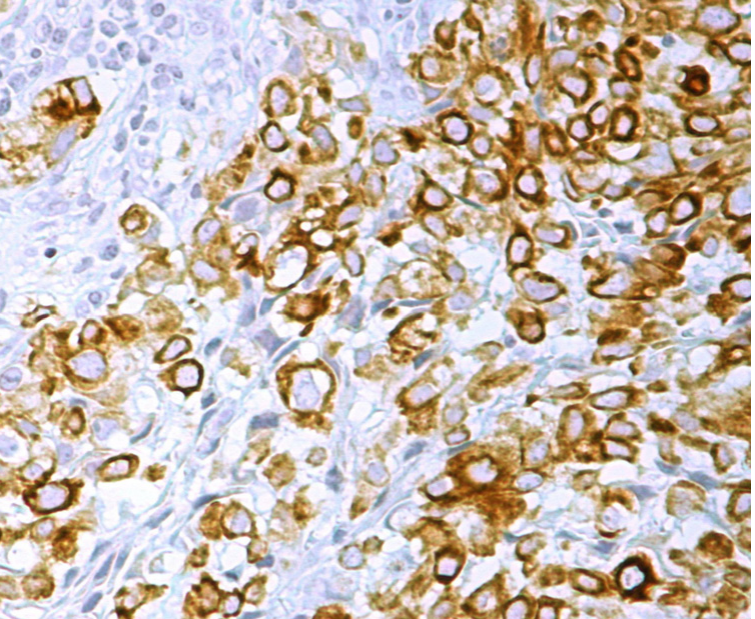
Immunohistochemical staining for S-100 showing cytoplasmic positivity. Magnification ×200.

The open biopsy specimen consisted of a rubbery, homogenous, white tissue with no attachment to the skin, implying deep location of the tumor. There were compact nests and fascicles with large rounded and spindle-shaped cells separated by connective tissue septa. The large rounded cells had pleomorphic nuclei and large amounts of clear cytoplasm while the spindle-shaped cells had palely staining eosinophilic cytoplasm. Mitoses were moderately numerous. There were areas of necrosis and hemorrhage and tumor spreading into the surrounding muscles and scapula. Immunohistochemical stains were performed with negative staining for CD68, actin and desmin antigens and with positive staining for tumor markers S-100 protein, HMB-45, NSE, EMA, cytokeratins and myosin. Histochemical stains for melanin presence were negative. Pathologic findings were compatible with a CCSTA with osseous extension into the lower scapula (Figure 4, 5).

After three days a wide excision was carried out and the tumor was removed together with the two thirds of the left scapula and the overlying soft tissues. The remaining bone and soft tissue margins were widely free of tumor. Macroscopic and pathologic examination of the removed mass confirmed the primary diagnosis.

Postoperatively the patient started on a chemotherapy protocol (three courses) consisting of ifosfamide, vincristine and epirubicin. The patient had no local recurrences but he developed lung metastases after 5 months. Despite chemotherapy, he died 10 months later with progressive lung disease.

## Discussion

CCSTA is an aggressive rare soft-tissue tumor with approximately 300 cases totally reported in the English literature [3]. It's a slowly growing tumor often with a long period from the first symptom to diagnosis (averaged 2 years) and seems to have no specific sex predilection. CCSTA can occur in patients of any age. The median age of the patients at the time of diagnosis is 29 years old [4]. In our case the interval between the presentation and the treatment was 4 months suggesting a fast growing tumor.

Commonly the presenting symptom is a painless mass or swelling. Pain and/or tenderness above the tumor site may be found in 33–50% of all the patients suffered from CCSTA. Typically CCSTA has the appearance of a palpable deep-located soft tissue mass bound to an adjacent tendon or aponeurosis. The overlying skin may occasionally appear with discoloration [4,5]. Intralesional calcifications are rarely seen and primary [2,6,7] or secondary [8,9] bone invasion by this tumor is very rare.

Frequently it arises in the extremities with a predilection for the lower limbs (78–97%). Both foot and ankle are the commonest sites of tumor appearance accounting 33–65% of all cases. Next most common sites are the knee, thigh, hand, forearm, elbow and shoulder in descending order of frequency; rarely the tumor arises in the head, neck or trunk [5,10,11].

Primary CCSTA overlying the scapular region, as in our case, is very uncommon with only a few case reports have been described [5,12]. This tumor has an unclear histological origin; there are many theories concerning histogenesis of CCSTA such as synovial origin, neural crest derivation or being an atypical malignant melanoma [5,12]. Tsuneyoshi *et al *[12] subdivided CCSTA into synovial and melanotic types based of melanin presence and histological and cytological differences.

Usually CCSTA has the appearance of a lobular or multinodular, circumscribed or encapsulated mass with a gray-white appearance on cut surface. Hemorrhage and necrosis may be present in large tumors. Intralesional calcifications are rarely seen [13]. Histologically, CCSTA is composed of round and/or fusiform cells arranged in nests separated by fibrocollagenous tissue. These cells have a relatively uniform appearance of round to ovoid vesicular nuclei with prominent nucleoli and with clear (in two-thirds of tumors perhaps due to intracytoplasmic glycogen) or eosinophilic cytoplasm. A small number of giant cells with more than 12–15 nuclei may be seen [14] but there were not wreath-like giant cells in our case. Fibrous tissue septa are often contiguous with the fibers of the involved tendons and aponeuroses.

Immunohistochemically, the tumor cells are usually positive for S-100 protein, HMB-45 and vimentin and/or microphthalmia transcription factor [14]. Cytokeratins (low molecular weight) may be found in the tumor cells [15]. Usually intracellular melanin is scanty[14] although many reported series refer that about 50–75% of CCSTA contain variable amounts of melanin which can be identified by melanin stains [4,5,11,12]. Ultrastructurally melanosomes may be present [14]. In our case the tumor did not contain melanin.

CCSTA is associated with chromosomal abnormalities consisting of changes in chromosome number and chromosome rearrangements [9,14,16]. Cytogenetic analysis of CCSTA identifies abnormalities in most chromosomes. A t(12;22)(q13;q12) translocation may be found in 75% of cases with CCSTA by karyotype analysis. Many authors suggest that this translocation seems to be pathognomonic for CCSTA [9,17].

Distinction between CCSTA and metastatic malignant melanoma may have diagnostic difficulties. Both tumors have similar histological features. However tumor presentation (especially location, cutaneous involvement, primary melanoma elsewhere) and histological features help to make a differential diagnosis between these tumors [14]. Both CCSTA and malignant melanoma are immunohistochemically positive for S-100 protein (a marker for tumors derived from neural crest) and HMB-45 (a marker identifying tumors associated with melanoma) [18] suggesting that these tumors have a close relationship. Primary and metastatic melanoma usually has a different genetic profile. The t(12;22)(q13;12) translocation has not yet been identified in melanoma [9,14]. Recent reports refer that there is a high incidence of activating mutations in the kinase domain of the BRAF gene in malignant melanoma of the skin while in CCSTA the BRAF mutations are rare [19,20].

The treatment of choice for patients with CCSTA is wide excision of the tumor or amputation with excision of regional lymph nodes. Adjuvant radiotherapy and/or aggressive multiagent chemotherapy seem to be ineffective but probably could be given [3-5,21,22]. Surgical excision, adjuvant radio- or chemotherapy or a combination of these three treatments seems to have no advantage of one therapy over another [4,21]. Our patient was submitted to surgical excision and chemotherapy and yet died after only ten months.

The patients with CCSTA have a variable unpredictable course. Despite treatment the overall prognosis is poor and subsequent wide spread dissemination of disease lead patients with CCSTA to death. The 5-year survival rate with radical excision and adjuvant chemotherapy and/or radiotherapy ranged from 60 to 67% and the 10-year survival rate is about 33% [4,5,10,11]. The incidence of local recurrence after primary surgical treatment is very high. Enzinger [1] reports incidence of 84% of first local recurrence (with 1–10 subsequent local recurrences), while in the series reported by Pavlidis [5] 5 of six cases developed local recurrence in 3 to 9 months.

Usually there is a median interval time between the primary treatment and first local recurrence ranged from 6 months to 1 year [11,12]. Therefore primary wide surgical excision of the tumor is warranted to decrease local recurrences. In our case no local recurrence was present after ten months.

The most common sites of metastases are the regional lymph nodes and the lungs and less common skin, bones, liver, heart and brain [2,5,11]. 60–70% of patients with CCSTA develop metastases at a mean time interval of 18 months to 6 years [1,5]. Perhaps preoperative duration of symptoms, tumor size, mitotic index or vascular invasion may not predict survival in these patients [5]. Some authors believe that the size of tumor defines a better or worse survival. Tumors greater than 5 cm have a worse survival and they warrant more aggressive local surgical treatment being at high risk of distant metastases [3,21] while in Deenik series [22] patients with tumor size less than 2 cm had a better survival.

## Competing interests

The author(s) declare that they have no competing interests.

## Authors' contributions

**CJK**: Preparation of manuscript, operation, **VGG**: Preparation of manuscript, operation, collection of clinical data and review of the literature, **AG**: Collection of data, carried out the pathologic findings **DJV**: Proofreading of manuscript, operation, collection of clinical data; **ES**: carried out the pathologic findings, proofreading of manuscript

All Authors read and approved the final manuscript.
